# The control of CD4^+^CD25^+^Foxp3^+ ^regulatory T cell survival

**DOI:** 10.1186/1745-6150-3-6

**Published:** 2008-02-27

**Authors:** Pushpa Pandiyan, Michael J Lenardo

**Affiliations:** 1 Laboratory of Immunology, National Institute of Allergy and Infectious Diseases, National Institutes of Health, Bethesda, MD, 20892, USA

## Abstract

CD4^+^CD25^+^Foxp3^+ ^regulatory T (T_reg_) cells are believed to play an important role in suppressing autoimmunity and maintaining peripheral tolerance. How their survival is regulated in the periphery is less clear. Here we show that T_reg _cells express receptors for gamma chain cytokines and are dependent on an exogenous supply of these cytokines to overcome cytokine withdrawal apoptosis *in vitro*. This result was validated *in vivo *by the accumulation of T_reg _cells in Bim^-/- ^and Bcl-2 tg mice which have arrested cytokine deprivation apoptosis. We also found that CD25 and Foxp3 expression were down-regulated in the absence of these cytokines. CD25^+ ^cells from Scurfy mice do not depend on cytokines for survival demonstrating that Foxp3 increases their dependence on cytokines by suppressing cytokine production in T_reg _cells. Our study reveals that the survival of T_reg _cells is strictly dependent on cytokines and cytokine producing cells because they do not produce cytokines. Our study thus, demonstrates that different gamma chain cytokines regulate T_reg _homeostasis in the periphery by differentially regulating survival and proliferation. These findings may shed light on ways to manipulate T_reg _cells that could be utilized for their therapeutic applications.

This article was reviewed by: Avinash Bhandoola, Fred Ramsdell (nominated by Juan Carlos Zuniga-Pflucker) and Anne Cooke.

## Background

CD4^+^CD25^+^Foxp3^+ ^T_reg _cells are a subset of lymphocytes having an anergic phenotype as shown by their absence of proliferation and production of IL-2 upon TCR stimulation. [1,2] They have been shown to suppress various inflammatory and autoimmune responses in mice and humans. Absence of this population of T cells causes an acute autoimmune condition called Immune dysregulation Polyendocrinopathy Enteropathy X-linked syndrome (IPEX) in humans and fatal autoimmune manifestations in mice [3-7]. T_reg _cells cause cytokine deprivation death by consuming cytokines from CD4 T cells to cause suppressive apoptosis. This is probably one of the default mechanisms of how T_reg _cells operate in the close vicinity of CD4 T cells [8]. Evidence show that self-peptides are important for homeostatic expansion of T_reg _cells in the periphery [9]. Despite the abundant availability of self-peptides, the frequency of T_reg _cells is always 10–15% of CD4^+ ^population. Increase or decrease in T_reg _numbers would result in immune imbalance as evidenced by suppressive effects of T_reg _cells on other immune cells. However, parameters controlling T_reg _cells and their survival maintaining the normal T_reg _numbers *in vivo*, remain unclear [10,11]. FAS and TCR restimulation mediated death constitute two of the major mechanisms regulating T cell survival and homeostasis [12-14]. However, T_reg _cells are shown to be resistant to these active forms of death [15,16]. IL-2 is shown to be a major survival factor of T_reg _cells, but the role of other cytokines is unknown[17]. One study proposes the role of gamma chain cytokines in regulating the suppressive potential of human T_reg _cells [18]. However, the direct contribution of these cytokines in the survival of T_reg _cells remains undocumented. Exploiting T_reg _cells for therapeutical applications demands a complete understanding of their survival mechanisms *in vitro *and *in vivo*. Here we show evidence that common γ chain cytokines play a major role in T_reg _survival in the periphery.

Common chain (γc) cytokines such as IL-2, IL-4, IL-7, IL-9, IL-15 and IL-21, bind to multimeric receptors that share the common γ chain (γ_c_) [19-26]. Common γc is a critical part of the cytokine receptors that confers the ability of as γc cytokines to activate MAP kinase and PI3 kinase signaling, leading to anti-apoptotic and proliferation signals in lymphocytes [24,27]. For example, IL-2 binds to IL-2R complex consisting of IL-2R-α, which possesses a short cytoplasmic domain. IL-2R-α binds IL-2 only with low affinity and does not recruit intra cytoplasmic signaling molecules. β chain (IL-2/15Rβ), shared by the IL-15 receptor stimulates downstream signaling pathways. However, γc is the most crucial component of the IL-2 receptor complex, raising its binding affinity for IL-2 and thus initiating a potent IL-2 signaling [28]. γc chain cytokines are pleiotropic soluble factors crucial for lymphocyte generation, survival and homeostasis [29]. Defects in γc signaling components result in impaired B, T, and natural killer (NK) cell development, leading to severe combined immunodeficiency in humans and mice [30,31]. The roles of IL-7 and IL-15 in the homeostasis of naïve CD4 and memory CD8 cells respectively, are well documented [21,24,32,33]. The importance of IL-2 in lymphocyte homeostasis is shown by a severe autoimmunity in mice deficient in IL-2 signaling components [34,35]. The autoimmune phenotype observed in these mice has been attributed to the loss of cell death mechanism mediated by FAS and absence of T_reg _cells in these mice [36,37]. Besides maintaining the homeostasis of naïve and memory cells, γc-cytokine signaling plays an important role during differentiation of activated T cells *in vivo *[25]. How γc-cytokines impact T_reg _cells *in vivo *is not well studied. The absence of T_reg _cells in γc-knockout mice seems to suggest that the common gamma chain signaling is important for the development of T_reg _cells. However, whether these cytokines influence the peripheral survival and expansion of T_reg _cells is not known. Here we show that gamma chain cytokines are crucial for maintaining the T_reg _cells in the periphery without which they undergo apoptosis.

## Methods

### Mice

BALB/c, C57BL/6, Bim^-/- ^mice and Scurfy mice were purchased from Jackson Laboratories. 129 Foxp3-eGFP transgenic mice were purchased from Taconic farms. CB-17 scid mice were also purchased from Charles River Laboratories. All mice were maintained in NIAID animal facility and cared for in accordance with institutional guidelines.

### Reagents and antibodies

Purified anti-CD3 (145-2C11), purified anti-CD28 (37.51), anti-CD25 (3C7), biotin-conjugated anti-CD25 (7D4), fluorescein isothiocyanate-conjugated anti-CD4 (GK1.5), phycoerythrin-conjugated anti-CD25 (PC61), unconjugated, allophycocyanin- or phycoerythrin-conjugated anti-IL-2 (JES6-5H4), anti-IL-4 (11B11) and anti-IL-4Rα (mIL4R-M1) are from BD Biosciences. Anti-Foxp3 (FJK-16S) and anti-IL-7Rα (A7R34) are from eBiosciences. The anti-FITC Multisort kit, IL-2 secretion assay kit and anti-biotin microbeads were from Miltenyi Biotec. The IL-2 Quantikine enzyme-linked immunosorbent assay (ELISA) kit and recombinant mouse IL-2, IL-7, IL-4, IL-15 and IL-21 were purchased from R&D Systems. Cell cultures were performed in complete RPMI 1640 medium (Bio-Whittaker) supplemented with 10% (vol/vol) FCS, 100 U/ml of penicillin, 100 μg/ml of streptomycin, 2 mM glutamine, 10 mM HEPES, 1 mM sodium pyruvate and 50 μM β-mercaptoethanol.

### Cell purification

Splenocytes were harvested from 5 to 12 week old mice. Erythrocytes were osmotically-lysed using Ack lysing buffer (Bio Whitaker) and single cell suspensions were incubated with FITC-conjugated anti-CD4 and biotin-conjugated anti-CD25 followed by incubation with anti-FITC microbeads. CD4^+ ^T cells were then purified by magnetic isolation using the Auto MACS sorter (Miltenyi Biotec). For isolation of CD4^+^CD25^+ ^T_reg _cells, after releasing the beads, the purified CD4^+ ^T cell suspension was incubated with α-biotin microbeads followed by separation using the Auto MACS. In all the experiments 90 to 95% of these cells were positive for CD4 and CD25. The negative fractions were depleted of CD25^+ ^cells to obtain CD4^+^CD25^- ^cells.

### T cell death and co-culture assays

T_con _cells (6 × 10^4^) or T_reg _were cultured in U-bottom 96-well plates in the presence of soluble 0.75 μg/ml α-CD3 and 3 μg/ml α-CD28 for 3–4 days. Death was measured by flow cytometry after 3 days. For co-culture assays, CD4^+^CD25^- ^responder T cells (T_resp_) (3 × 10^4^) were cultured in U-bottom 96-well plates with T_con _(CD4^+^CD25^-^) (3 × 10^4^) or T_reg _(CD4^+^CD25^+^) in the presence of soluble 0.5–0.75 μg/ml α-CD3 and 3–4 μg/ml α-CD28 for 2–4 days. T_reg _or T_con _cells were used in the co-culture with responders directly in U-bottom 96-well plates. T_resp _cells were CFSE-labeled to distinguish them from T_con _or T_reg _cells in co-culture. Proliferation was also assayed by CFSE dilution. Cell death analyses of CFSE^+ ^responders were performed based on forward scatter and propidium iodide staining. All flow cytometry analyses assessing cell death were performed with events acquired at constant time, in order to count the events. The percentage of survival (Survival (%)) in all analyses is the percentage of cells that FSC^high ^and PI^-^. When indicated, IL-2 (1000 U/ml), IL-7 (20 ng/ml), IL-4 (20 ng/ml), IL-15 (20 ng/ml), IL-21 (20 ng/ml) was added. For IL-2 blocking experiments, CD4^+^CD25^- ^cells were isolated and 6 × 10^4 ^cells were stimulated with anti-CD3 and anti-CD28 in the presence of isotype control or cytokine blocking antibodies, 10 μg/ml each, and cultured in 96-well U-bottomed plate.

### Electron microscopy

T_con _or T_reg _cells isolated from cultures were washed with PBS twice, fixed with fixation buffer containing Glutaraldehyde and Sodium Cacodylate. Fixed cells were pelleted and sent to electron microscopy facility at SAIC-Frederick, Inc. for imaging and analyses.

## Results and Discussion

### Gamma chain cytokines are essential for the survival of T_reg _cells *in vitro*

**Figure 1:**
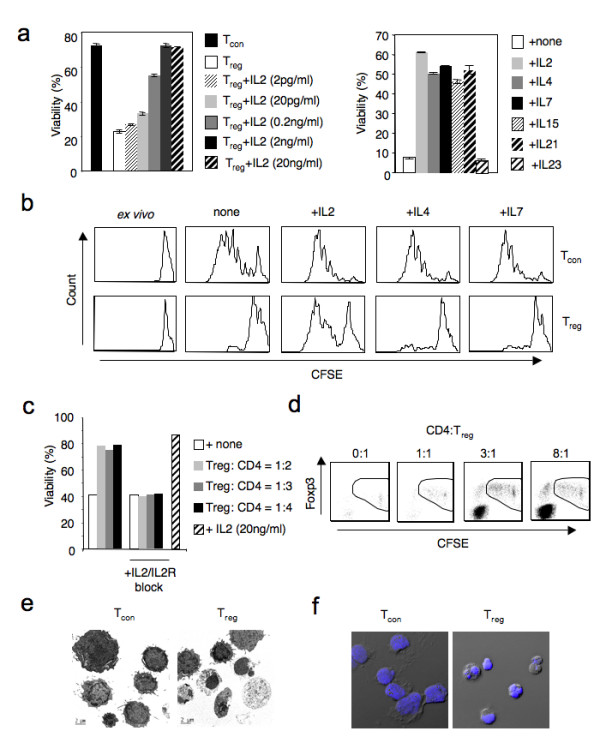
**Gamma chain cytokines rescue T_reg _cells from apoptosis *in vitro***. **(a) **Viability of T_con _or T_reg _cells stimulated for four days with soluble anti-CD3 and anti-CD28. IL-2 was added at indicated concentrations in T_reg _cultures at the beginning of stimulation (left panel). Percentages of events in the live gates (PI^neg ^and FSC^high^) in flow cytometric analyses are shown. Viability of T_reg _cells stimulated as in '*a*'. Indicated cytokines were added at 20 ng/ml in T_reg _cultures at the beginning of stimulation (right panel). (**b**) Proliferation of CFSE labeled T_con _or T_reg _cells that were isolated *ex vivo *or stimulated as in '*a*' with or without indicated cytokines, each at 20 ng/ml concentration. (**c**) Viability of CFSE labeled T_reg _cells that were stimulated as in 'a' and cultured without or with indicated numbers of conventional CD4^+ ^T cells. CFSE labeling was done to distinguish T_reg _cells and conventional CD4^+ ^T cells in the cultures. IL-2 and IL-2 receptors were blocked using blocking antibodies added at 10 μg/ml each, in the beginning of the stimulation. Data from (**a-c**) represent 3 independent experiments (**d**) Proliferation of CFSE labeled Foxp3^+ ^T_reg _cells that were stimulated as in 'a' and cultured without or with CD4^+ ^T cells at indicated ratios. Contaminating Foxp3^- ^population in CFSE labeled T_reg _population is excluded in the analyses. Electron micrographs (**e**) or confocal microscopy analyses (**f**) of 2 or 3-day stimulated T_con _or T_reg _cells showing apoptotic T_reg _cells at different stages as defined by condensed, shrunken nuclei. (Blue = nuclei stained by 6-diamidino-2-phenylindole (DAPI) in (**f**).

To examine the survival of T_reg _cells *in vitro*, we used magnetically sorted CD4^+^CD25^-^Foxp3^- ^T cells (T_con_) or CD4^+^CD25^+ ^(T_reg_) cells and cultured with soluble anti-CD3 and anti-CD28 for 72–96 hours. We measured the frequency of surviving cells based on forward scatter and propidium iodide (PI) staining and flow cytometry analyses. There was a dramatic T_reg _cell death (75–90%) in the absence of IL-2 and increasing doses of IL-2 rescued them in a concentration dependent manner (Fig. 1a, **left panel**). T_con _cells survived well without exogenous IL-2 in the cultures, presumably because they produce IL-2 themselves (Fig. 1a, **left panel**). To test the effect of other γ_c _cytokines, we added IL-2, IL-4, IL-7, IL-15 or IL-21 at 20 ng/ml concentration during the beginning of stimulation in T_reg _cultures. We observed that the presence of the cytokines rescued T_reg _death, with IL-2 having the strongest pro-survival function (Fig. 1a, **right panel**). On the other hand, IL-23 a non-γ_c _cytokine did not have an effect on the survival of T_reg _cells (Fig. 1a, **right panel**). Carboxyfluorescein succinimidyl ester (CFSE) labeling of the cells showed that in the absence of cytokines, few T_reg _cells that remained in the culture did not undergo proliferation whereas T_con _cells proliferated vigorously (Fig. 1b). IL-2 induced proliferation in T_reg _cells whereas IL-4, IL-7, IL-15, IL-21 had minimal effect on proliferation even at the excessive concentration of 20 ng/ml (Fig. 1b, **data not shown**). However, the possibility that the combination of some or all γ_c _cytokines such as IL-4, IL-7, IL-15 and IL-21 could initiate proliferation in Treg cells is not excluded. As a control for CFSE staining, un-stimulated T_reg _cells isolated *ex vivo *is shown (Fig. 1b). Next, we hypothesized that T_con _cells producing IL-2 might also serve as IL-2 source and support the survival and proliferation of T_reg _cells in cultures. Therefore we stimulated the CFSE labeled T_reg _cells with increasing numbers of CD4^+ ^T cells and analyzed their death after 72 hours. As expected, T_reg _cells died less and also proliferated in the presence of conventional CD4^+ ^T cells (Fig. 1c). Even though conventional CD4^+ ^T cells themselves died in the presence of T_reg _cells at CD4:T_reg _in 1:1 ratio, their viability was only mildly affected at 3:1 and 8:1 ratios. To test if T_con _induced survival was mediated by IL-2, we blocked IL-2 using a blocking antibody and found that the survival frequencies of T_reg _cells fell back to basal levels even in the presence of CD4^+ ^T cells (Fig. 1d). However, the direct effect of blocking IL-2 in T_con _cells cannot be ruled out. The number of proliferating T_reg _cells was directly proportional to the numbers of viable CD4^+ ^T cells (Fig. 1d). This is in accordance with our previous findings that T_reg _cells consume cytokines from conventional CD4 T cells, and in the process, suppress them [8]. Transmission electron microscopy and confocal microscopy analyses of T_reg_cells in the absence of cytokines showed condensed nuclei and membrane blebbing, the characteristic features of apoptosis (Fig. 1e and 1f). Thus, it is evident that T_reg _cells undergo apoptosis in the absence of cytokine signaling *in vitro*. Our findings corroborate the observation that T_reg _cells are absent in γ_c_-knockout mice, implying that gamma chain signaling is important not only for the development but also for the survival of T_reg _cells in the periphery. Our observation that gamma cytokines besides IL-2 can support the survival of T_reg _cells potentially explains previous observation that shows only a reduced frequency and not a complete absence of Foxp3^+ ^cells in CD25 deficient mice [38]. In the absence of IL-2-R signaling, the combination of other γ_c _cytokines could also induce both survival and homeostatic proliferation of T_reg _cells in the periphery.

### T_reg _cells express cytokine receptors *in vitro*

**Figure 2:**
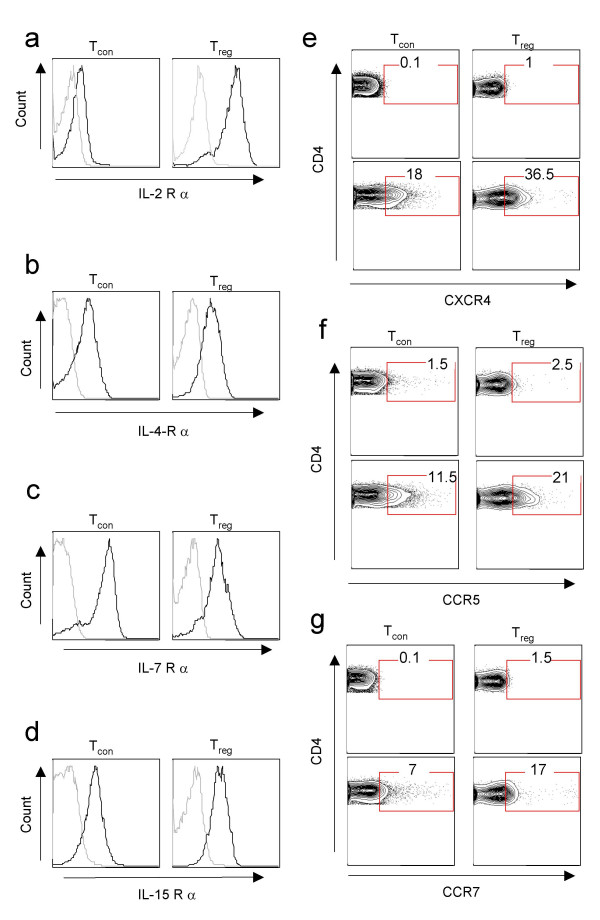
**T_reg _cells express cytokine and chemokine receptors *ex vivo***. Expression of cytokine receptors on splenocytes isolated from Foxp3 GFP mice in which Foxp3 is expressed as a fusion protein with GFP. Grey curves indicate unstain controls. (**a-d**). Expression of indicated cytokine receptors on GFP^- ^(T_con_) or GFP^+ ^(T_reg_) of splenocytes isolated *ex vivo*. (**e-g**). Expression of indicated or chemokine receptors of unstimulated T_reg _cells (upper panels) or 3-day α-CD3 and α-CD28 stimulated T_reg _cells (lower panels). Results are representative of two independent experiments.

To determine if T_reg _cells express the receptors for cytokines in addition to IL-2R-α CD25, splenocytes were isolated from Foxp3-eGFP mice and stained for cytokine receptors such as IL-4R-α (CD124), IL-7R-α (CD127) and IL-15R-α *ex vivo*. We found that T_reg _express receptors for these cytokines (Figs. 2a,b,c,d). We could not detect IL-21R-α expression on T_con _and T_reg _cells, possibly due to the absence of good detecting antibody for mouse IL-21R-α (data not shown). We also found that T_reg _cells showed an upregulation of chemokine receptors such as CXCR4, CCR5 and CCR7 up on α-CD3 and α-CD28 stimulation (Fig. 2e,f and 2g, **upper and lower panels**). These chemokine receptors on T_reg _cells are likely important for the T_reg _cells to be recruited to the chemokine and possibly also cytokine producing cells at the sites of inflammation.

### T_reg _cells die due to cytokine deprivation *in vivo*

**Figure 3:**
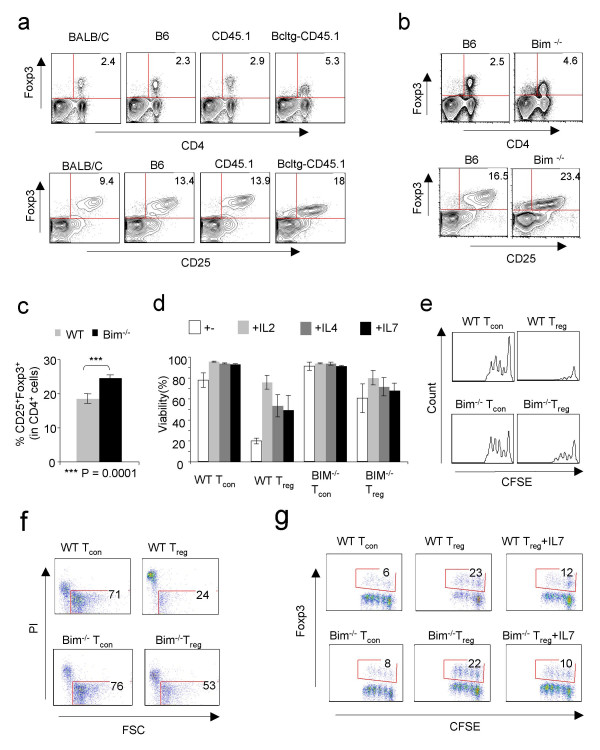
**T_reg _cells are susceptible to cytokine deprivation apoptosis *in vivo***. (**a**) Frequency of CD4^+ ^Foxp3^+ ^(upper panels) or CD25^+^Foxp3^+ ^(lower panels) in splenocytes isolated from WT, Bcl-2 tg mice *ex vivo*. **(b) **Splenocytes of WT or Bim^-/- ^mice showing the frequency of CD4^+ ^Foxp3^+ ^(upper panel) or CD25^+^Foxp3^+ ^(lower panel) T_reg _cells *ex vivo *(**c**) Mean percentage of T_reg _cells in spleens of WT (grey bar) or Bim^-/- ^mice (solid black bar) (n = 3–5, n = number of mice). Data represent two independent experiments. (**d**) Viability of T_con _or T_reg _cells from WT or Bim^-/- ^mice stimulated for 3 days with soluble anti-CD3 and anti-CD28. Cytokines were added at indicated 20 ng/ml concentrations in cultures at the beginning of stimulation. Percentage of events in the PI^neg ^and FSC^high ^live gates are shown. Histograms of CFSE dilution of live T_resp _cells (**e**) and level of apoptosis in T_resp _cells (**f**) from WT mice co-cultured with T_con _cells or T_reg _cells from WT (upper panels) or Bim^-/- ^(lower panels) mice. (**g**) Dot plots showing Foxp3 expression and CFSE dilution in live T_resp _cells co-cultured with WT (upper panels) or BIM^-/- ^(lower panels) T_con _or T_reg _cells stimulated as in '*d*' for 4 days in the presence or absence of IL-7 (20 ng/ml).

To further assess the role of apoptosis in regulating T_reg _cells *in vivo*, we determined the role of B cell lymphoma-2 (Bcl-2) protein in T_reg _death. To this end, we measured the frequency of CD25^+ ^Foxp3^+ ^cells in Bcl-2 transgenic mice in CD45.1 background. We found that there was an increased percentage of T_reg _cells in these mice (Fig. 3a). The specific role of Bcl-2 interacting member (Bim) protein in mediating cytokine deprivation apoptosis is well documented [39]. Therefore, we tested the frequency of T_reg _cells in Bim deficient mice. Surprisingly, we also found that there was an increased accumulation of T_reg _cells in the spleens of Bim^-/- ^mice (Fig. 3b and 3c). Furthermore, when we stimulated Bim^-/- ^CD4^+ ^T_reg _cells *in vitro*, we found that they had increased resistance to death in the absence of cytokines (Fig. 3d). However, their suppressive phenotype remained intact in the absence of Bim. They induced a partial suppression in proliferation and cell death of CFSE labeled T_resp _cells that were co-cultured with them as compared to those with T_con _cells (Fig. 3e and 3f). In addition to causing cell death in CD4^+ ^T_resp _cells, both WT T_reg _cells and BIM^-/- ^T_reg _cells were able to induce Foxp3 in conventional CD4^+ ^T cells in co-cultures (Fig. 3g). The relevance of Foxp3 induction in T_resp _cells due to the presence of T_reg _cells is unknown presently. However, in the presence of T_reg _cells and IL-7, the frequency of induced Foxp3+ cells seems to be diminished, probably due to an increased proliferation of non Foxp3^+ ^CD4^+ ^T_resp _cells as compared to induced Foxp3^+ ^CD4^+ ^T_resp _cells in the presence of IL-7.

### Gamma chain cytokines maintain CD25 and Foxp3 expression in T_reg _cells

**Figure 4:**
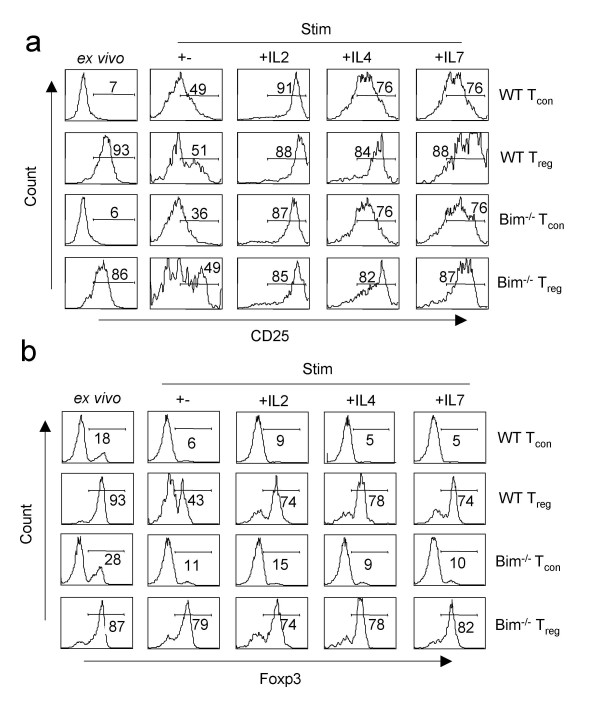
**Gamma chain cytokines maintain CD25 and Foxp3 expression in T_reg _cells**. Histograms of CD25 expression (**a**) or Foxp3 (**b**), of live T_con _or T_reg _cells from WT (two upper panels) or Bim^-/- ^(two lower panels) mice stimulated for 3 days with soluble anti-CD3 and anti-CD28. Cytokines were added at indicated 20 ng/ml concentrations in cultures at the beginning of stimulation. Gates show CD25^high ^(**a**) or Foxp3^high ^(**b**) cells.

Because CD25 and Foxp3 are important for the function for T_reg _cells, we tested the influence of gamma chain cytokines on the expression for these molecules. T_reg _cells stimulated without any cytokine showed a substantial down-regulation of the CD25 expression whereas CD25 up-regulation was normal on T_con _cells (Fig. 4a, **two upper panels**). In the presence of cytokines however, CD25 expression was maintained at high levels both on T_con _cells and T_reg _cells (Fig. 4a, **two upper panels**). CD25 down regulation was only partial on BIM^-/- ^T_reg _cells in the absence of cytokines, but was further up-regulated in the presence of cytokines (Fig. 4a, **two lower panels**). We also found that in WT T_reg _cells, Foxp3 was down regulated in the absence of cytokines whereas Foxp3 levels remained high in Bim^-/-^T_reg _cells upon TCR stimulation (Fig. 4b). Cytokines maintained high levels of Foxp3 expression both in WT and Bim^-/- ^T_reg _cells (Fig. 4b). On the other hand, WT and Bim^-/- ^T_con _population had only few Foxp3^+ ^cells upon TCR stimulation, and was not up-regulated in the presence of cytokines. These findings demonstrate that cytokines are crucial not only for the survival but also for maintaining the cardinal features of T_reg _cells i.e the expression of CD25 and Foxp3. We believe that T_reg _cells lose the expression of Foxp3 due to the initiation of death signals in the absence of cytokines because it did not occur in the absence of death in Bim^-/- ^T_reg_cells.

### Foxp3 dictates the cytokine dependence in T_reg _cells

**Figure 5:**
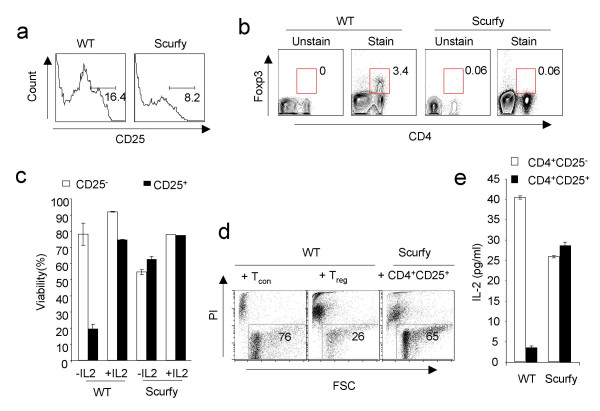
**Foxp3 repression of IL-2 determines the IL-2 dependence in T_reg _cells**. (**a**) Expression of CD25 on splenocytes isolated from WT or Scurfy mice *ex vivo *(gated on CD4^+ ^T cells). (**b**) Expression of intracellular Foxp3 in splenocytes from WT or Scurfy mice showing the frequency of T_reg _cells. (**c**) Viability of T_con _or CD25^+ ^cells from WT or Scurfy mice stimulated for 3 days with soluble anti-CD3 and anti-CD28 with or without IL-2 added at 20 ng/ml concentration. (**d**) Viability of T_resp _cells from WT mice stimulated for 3 days with soluble anti-CD3 and anti-CD28 and co-cultured with T_con _cells or CD25^+ ^cells from WT or Scurfy mice. (**e**) ELISA quantification of IL-2 in T_con _(open bars) or CD25^+ ^(solid bars) cells from WT or Scurfy mice, stimulated for 3 days with soluble anti-CD3 and anti-CD28.

Scurfy mice carry a mutation in Foxp3 and succumb to a fatal autoimmune syndrome. We tested whether a strong dependence of cytokines was a characteristic feature of all *ex vivo *isolated CD25^+ ^cells in general. We found that these Scurfy mice harbored CD4^+ ^CD25^+ ^cells that are presumably activated CD4^+ ^T cells owing to the autoimmune condition of the mice (Fig. 5a). However, the CD25^high ^cells that represent the T_reg _population was reduced from 16% to 8% in CD4^+ ^population as compared to WT mice (Fig. 5a). However, there was a complete absence of Foxp3^+ ^cells in these mice (Fig. 5b). To investigate whether Foxp3 is important in T_reg _cells for the extreme cytokine dependence for their survival, we isolated CD25^+ ^cells from the Scurfy mice and tested their survival in the presence or in the absence of IL-2. We found that isolated Scurfy T_con _cells, when stimulated in cultures had an impaired survival as compared to WT T_con _cells. Surprisingly, however we found that Scurfy CD25^+ ^cells survived as well as Scurfy T_con _cells even in the absence of IL-2 whereas WT T_reg _cells died substantially (Fig. 5c). Because of the lack of dependence on IL-2, we hypothesized that Scurfy CD25^+ ^cells might not suppress other conventional T cells. To test this tenet, we co-cultured the CD25^+ ^cells from WT mice or Scurfy mice with CFSE labeled CD4 T cells and measured the suppressive death after 3 days. Interestingly, we observed that responding T_resp _cells underwent death in the presence of CD25^+ ^cells from WT mice and not with CD25^+ ^from Scurfy mice (Fig. 5d). It is likely that CD25^+ ^cells from Scurfy mice produce cytokines due to the absence of Foxp3, which is why they do not depend on IL-2 added exogenously. To validate this theory, we stimulated WT T_reg _cells and scurfy CD25^+ ^cells with anti-CD3 and anti CD28 and measured IL-2 in the supernatants after 3 days. We found that while WT T_reg _cells did not produce IL-2, Scurfy CD25^+ ^cells produced as much cytokine as Scurfy CD4^+ ^CD25^- ^cells approaching the level of cytokine produced by WT CD4^+^, CD25^- ^cells (Fig. 4e). This data is consistent with the previous observations showing suppressive effects of Foxp3 on IL-2 production [40,41] and the effect of IL-2 on T_reg _homeostasis [11,42]. Together, we show here that Foxp3 represses cytokine production in T_reg _cells, which is why they are dependent on gamma cytokines from an external source for survival. Thus T_reg _cells have a self-regulatory mechanism through which their inability to produce cytokines instruct them to depend on cytokines and without the cytokines, the T_reg _cells are deleted.

## Conclusion

Taken together, our data have important implications in the understanding of behavior and regulation of T_reg _cells. Here, we demonstrate that Treg cells are highly susceptible to apoptosis in the absence of cytokines. This cytokine withdrawal apoptosis in T_reg _cells is substantially abolished by the γc cytokines *in vitro*. Our data also reveal that Bim^-/- ^mice accumulate higher frequencies of T_reg _cells showing the importance of cytokine withdrawal death in regulating peripheral T_reg _cells. Moreover, T_reg _cells from Bim^-/- ^mice do not depend on cytokines for survival *in vitro*. In addition to enhancing their survival, the γc cytokines also maintain CD25 and Foxp3 expression in T_reg _cells, thus maintaining their suppressive potential. Most importantly, our data show that Foxp3 appears to confer the inability to produce cytokines in T_reg _cells thus increasing their dependence to extra-cellular sources of cytokines for survival and function. We have described here a cytokine dependent homeostatic regulation mechanism of the T_reg _cells. Along with the self-peptides, the availability of γc cytokines probably keeps the T_reg _numbers in constant check thus maintaining both protective and regulatory arms of the immune system in balance. Thus, our study highlights the important role of γc cytokines in regulating T_reg _survival, opening new ways to manipulate T_reg _cells.

## Reviewers' comments

### Reviewer's report 1

Dr Avinash Bhandoola, University of Pennsylvania School of Medicine, Philadelphia

PA United States

I thought there was plenty of interesting new data in this work. I have a few very minor comments that should be simple to deal with, and do not need to be published. 1) I thought the abstract somewhat repetitious in places. It could be shortened. 2) The figure legends do not clearly explain 3b, particularly the bottom 2 panels. 3) I did not understand the relevance of the right-most panels in Fig. 3g (WT or Bim-/- Treg + IL-7), particularly when compared to the two preceding panels (WT or Bim-/- Treg). Is it referred to at all in the text, or otherwise explained?

#### Author response

*Relevant changes are made in the abstract and more clarifications are included in the figure legends according to reviewer's comments*.

### Reviewer's report 2

Fred Ramsdell, Associate Director, Zymogenetics, Seattle, WA 98102

Overall, the manuscript is well-written and concise. The connection between g-c receptor signaling and apoptosis – and the distinction between these cytokines and proliferation – is a significant finding and generally well supported by the data. To date, the bulk of studies on Treg survival/activity and cytokines has focused on IL-2, and the extension to other gamma-c receptor using cytokines provides a more comprehensive analysis of this biology.

Whilst the experiments in Fig 5 are an interesting attempt to address the function of Foxp3 with respect to cytokine dependence, the conclusions are not fully supported by the data. The abstract states that in scurfy mice, "Foxp3 increases their (CD25+ cells) dependence on cytokines by suppressing cytokine production in Treg cells." Whilst Foxp3 does appear to directly suppress cytokine production, it does so in any T cell and the major effect of lack of Foxp3 in scurfy mice would appear to be the absence of the T_R _lineage more broadly. Thus, this is not an appropriate way to test "whether Foxp3 is important in Treg cells for the extreme cytokine dependence for their survival" as these mice don't have Treg cells. Previous data has demonstrated that scurfy T cells do not express Foxp3, that they are CD25+ and that these cells do not have any Treg activity (Khattri, et. al.). In fact, these cells produce large amounts of IL-2 and many other cytokines. Importantly however, the cells remaining in scurfy mice do not appear to be in any way related to Treg cells. The data in the manuscript is consistent with data from Foxp3 transgenic mice in which Foxp3 levels are increased, but the actual number of Treg cells is decreased, as are their CD25 levels – perhaps due to Foxp3 inhibition of g-c derived (IL2 or other) secretion. This figure however does not seem necessary to me for the manuscript to be of interest.

#### Author response

*This data indicates that not all CD25^+ ^cells consume and depend on cytokine in vitro. In the absence of Foxp3 in Scurfy mice, CD25^+ ^cells do not depend on IL-2 and other cytokines and make cytokines themselves. Even though our data do not show that Foxp3 determines cytokine dependence directly, we feel that there is a strong implication that the presence of Foxp3 inversely correlates with cytokine production in Treg cells, based on the fact that Foxp3 expression is restricted to Treg cells in mice. The weakness in this experiment, and a point on which we agree with the reviewer, is that it is not clear that the CD25-expressing cells in the Scurfy mice are related to CD25-expressing Treg cells in WT mice. For example, if Treg cells are truly absent in Scurfy animals, then the CD25-expressing T cells could be from a completely different lineage of CD4+ T cells. In this case, we would be comparing different lineages and the results would not indicate a direct effect of FoxP3. Alternatively, it might be that the FoxP3-negative, CD25-expressing cells in Scurfy mice are cells that would otherwise would have become Tregs, then our data gives a better insight into the role of FoxP3. In either case, a cleaner experiment would be to perform a knockdown of FoxP3 in WT mouse Tregs and assess if they now produce cytokines at a normal level and are no longer susceptible to apoptosis in the absence of exogenous cytokines. We are working on executing this experiment in the future. However, the results in *Fig. 5*show that Foxp3 expression and function but not expression of cytokine receptors alone determines cytokine consumption. Sakaguchi and colleagues (Hori et al, 2003, Science) have shown that Foxp3 transduction alone converts normal T cell in to a Treg cell validating our finding that the function of Foxp3 is restricted to Treg cell. Therefore, we feel that *Fig. 5* is necessary for the manuscript*.

One further observation is that it appears to be very clear in Fig 3a/b that the amount of Foxp3 protein is substantially less in the Bcl-2 tg and Bim -/- Treg cells than in conventional cells (although this is less evident in Fig 4). Previous data has suggested that the absolute amount of Foxp3 can be a critical factor in regulating the amount of suppressive activity by Treg cells, and the functional data in Fig 4e/f would support this. But I am unclear why, in the model proposed, there might be less Foxp3 protein in cells from these animals and I would be interested to hear the author's speculation on this.

#### Author response

*We speculate that Treg cells in Bcl-2 tg and Bim-/- mice, do not die even when cytokine levels are less abundant resulting in accumulation of T_reg _cells. Therefore, the available cytokines are being shared by more T_reg _cells. Each T_reg _cell could potentially be exposed to lower amounts of cytokines, which possibly results in Foxp3 downregulation (because cytokines maintain Foxp3 expression)*.

##### Some minor points for consideration

Although correct as written, it might be more informative to indicate in the abstract that ANY g-c using cytokine protects Treg cells from apoptosis – although only IL2 appears to be capable of inducing proliferation. This distinction is one of the more salient features of the article. It seems appropriate to reference the work of Malek and colleagues (particularly Bayer, Yu and Malek, JI, 2007) when referring to previous studies on the role of IL-2 and Treg development (eg, in reference to Fig 1). In Figure 2, for panels e-g, please clarify the conditions for the upper versus lower histograms (presumably resting versus activated). I declare that I have no competing interests.

#### Author response

*The abstract has been rewritten reiterating the differential effects of gamma chain cytokines on survival and proliferation. The *Figure. 2* legend has been modified and the Malek reference is included as suggested by the reviewer*.

### Reviewer's report 3

Anne Cooke, University of Cambridge, Department of Pathology, Tennis Court Rd

Cambridge, CB21QP, United Kingdom

In this manuscript the authors have examined the role of common gamma chain (γc) cytokines in CD4^+ ^CD25^+ ^Foxp3^+ ^T cell survival. They clearly show that cytokines other than IL-2 that signal through the γc prevent apoptosis of T reg cells following stimulation with αCD3 and αCD28. This provides a nice explanation for the presence of T reg in CD25 deficient mice. While addition of exogenous γc cytokines enabled T reg survival, they are proposed individually not to be as effective as IL-2. This reviewer was unclear whether all the cytokines had been titrated to determine efficacy. Were the cytokines titrated fully and were doses greater than 20 ng/ml used?

#### Author response

*All cytokines were used at 20 ng/ml, which is an excessive amount in culture of 60,000 cells based on established biologically active concentrations*.

The link to apoptosis in Treg survival was nicely further substantiated by studies using T reg from Bcl-2 transgenic or BIM deficient mice. It appeared that there was some induction of Foxp3 expression in responding cells co-cultured with Treg. Was this TGFβ and/or cell contact dependent? The authors do not comment on the ability of IL-7 to reverse this.

#### Author response

*We did not test if the induction of Foxp3 in T_resp _cells was TGF-β dependent as it was not the focus of the current study. It is an interesting experiment for the future and we appreciate this suggestion. However, in the presence of T_reg _cells and IL-7, the frequency of induced Foxp3+ cells seems to be diminished, probably due to an increased proliferation of non Foxp3^+ ^CD4^+ ^T_resp _cells as compared to induced Foxp3^+ ^CD4^+ ^T_resp _cells in the presence of IL-7. We have included this comment in the last sentence of the relevant paragraph in the results section*.

The final observation that Foxp3 expression suppresses cytokine production in T reg cells is interesting and in line with the studies of others. It was somewhat surprising that the work of others was not mentioned and the data from this current submission not situated in the context of the studies by Sakaguchi and his colleagues (Ono et al (2007) Nature 446:685–689.) showing Runx1 interaction with FoxP3 and inhibition of IL-2 gene transcription as well as others. Rao and colleagues (Wu et al Cell 2006) had also previously predicted an effect of FoxP3/NFAT interaction on the transcription of several genes including IL-2. The manuscript would have been improved by including some discussion of these.

#### Author Response

*References and discussion are included as per the suggestions of the reviewer*.

## Competing interests

The author(s) declare that they have no competing interests.

## Authors' contributions

PP designed the study and performed the experiments under the supervision of MJL. The manuscript was written by PP and MJL.
